# A new species of *Rhytidognathus* (Carabidae, Migadopini) from Argentina

**DOI:** 10.3897/zookeys.247.3303

**Published:** 2012-11-30

**Authors:** Sergio Roig-Juñent, Julia Rouaux

**Affiliations:** 1 Laboratorio de Entomología. Instituto Argentino de Investigaciones de las Zonas Áridas (IADIZA, CCT CONICET Mendoza), CC 507, 5500 Mendoza, Argentina; 2 Departamento de Entomología. Museo de La Plata. Paseo del Bosque S7N, 1900, La Plata

**Keywords:** Migadopini, *Rhytidognathus*, New species, Male and female genitalia, Distribution

## Abstract

The Migadopini are a small tribe of Carabidae with 47 species that occur in South America, Australia, and New Zealand, in the sub-Antarctic areas. In South America, most of the genera inhabit areas related to sub-Antartic *Nothofagus* forest except two monogeneric genera, the Ecuadorian genus *Aquilex* Moret and the Pampean genus *Rhytidognathus* Chaudoir. These two genera are geographically isolated from the remaining five South American genera. New material of *Rhytidognathus* from the northeast of Buenos Aires province and from Entre Ríos province permits establishing that the previous records of *Rhytidognathus ovalis* (Dejean) for Argentina were erroneous and that it belongs to a new species. Based on external morphological characters and from male and female genitalia we describe *Rhytidognathus platensis* as a new species. In this contribution we provide illustrations, keys, habitat characteristics and some biogeographic considerations on the distribution of *Rhytidognathus*.

## Introduction

The Migadopini are a small tribe of Carabidae, with 16 genera and 47 species. This tribe was considered related to the Holarctic tribes Elaphrini and Loricerini (Jeannel 1938), and Loricerini (Maddison et al. 1999). Ball and Erwin (1969) considered that the characters shared with Loricerini are convergent and do not show an ancestral relationship. The most modern classification considers the Migadopini as constituting the subfamily Migadopinae, together with the tribe Amarotypini (Johns 2010).

The species of Migadopini are distributed over fragments of the austral Gondwana, called Paleantarctic by Jeannel (1938). These species occur in southern South America (eight genera with con 17 species) (Roig-Juñent 2004), one monotypic genus in the Andean region of northern South America (Moret 1989), four genera with seven species in Australia (Baher 2009) and four genera with 19 species in New Zealand and circum-Antarctic islands (including a new genus and several new species not yet described) (Johns 2010). The only complete revision of the tribe is that by Jeannel (1938). Later, for South America, Straneo (1969), Négre (1972), and Baher (1997; 1999) described new species or subspecies, Moret (1989) described a new genus and species and finally Roig-Juñent (2004) redescribed all the austral South American genera including male and female genitalia characters and developed a cladistic and biogeographic analysis of the genera. For Australia, Baher (2009) described a new genus with two species, and for New Zealand, Johns (2010) described 11 new species.

The number of species per genus is low. Of the 16 genera, eight are monospecific, four have two species and the most diverse in number of species is *Taenarthrus* Broun with 12 species (Johns 2010).

Migadopines constitute a characteristic element of the sub-Antarctic biota, and except some frequent species such as the South American *Migadops latus* (Guérin-Ménéville) the others are scarce in natural history collections, with just a few specimens of several species known. This is the case for the genus *Rhythidognathus* Chaudoir of which only 12 specimens are known: the holotype of *Rhythidognathus ovalis* (Dejean), nine more specimens from Uruguay, and two from Argentina. Of these last two specimens, one is lost, and we only have the account by Tremoleras (1931). Strange as well is the particular distribution of the genus *Rhytidognathus*, because it does not inhabit sub-Antarctic habitats, and its phylogenetically related genera are about 3000 km to the south.

Ecological studies conducted in the area of La Plata (Buenos Aires, Argentina) yielded the discovery of new specimens of *Rhytidognathus*, and particularly the capture of males allowed establishing that the previously cited species of *Rhytidognathus* from Argentina (Tremoleras 1931, Roig Juñent 2004) is not *Rhythidognathus ovalis* but instead a new species.

The objective of the present contribution is to describe this new species, including new data on its habitat, and discuss some biogeographic considerations.

## Material and methods

**Material examined.** The material is held in the following institutions: IADIZA: Instituto Argentino de Investigaciones de las Zonas Áridas (Mendoza, Argentina, Sergio Roig-Juñent); MACN: Museo Argentino de Ciencias Naturales “Bernandino Rivadavia” (Buenos Aires, Argentina, Arturo Roig-Alsina); MLP: Museo de La Plata (La Plata, Argentina, Analía Lanteri).

Dissection methods, measurements, and the terminology used follow previous revisions of Migadopini (Jeannel 1938, Moret 1989, Roig-Juñent 2004, Johns 2010).

Predictive species distribution models were built using the MAXENT program version 3.4.1 (Phillips et al. 2006), because MAXENT performed well with small sample sizes (Tognelli et al. 2009), which is the case of *Rhytidognathus*. Also because of the low number of known species localities, we performed the analysis at generic level.

### Rhytidognathus

Chaudoir, 1861

http://species-id.net/wiki/Rhytidognathus

#### Type species.

*Nebria ovalis* Dejean, 1831, by monotypy.

#### Redescription.

**Figure 1.**
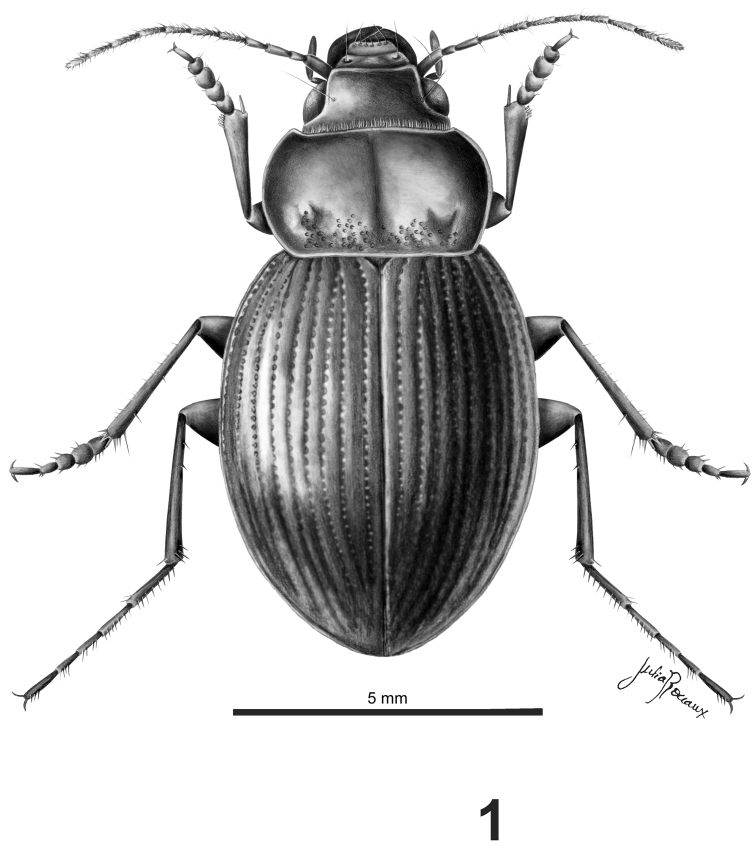
Dorsal aspect of male *Rhytidognathus platensis* (Scale = 5 mm).

*Habitus*.Body shape rounded, depressed (Fig. 1)

**Figures 2–7.**
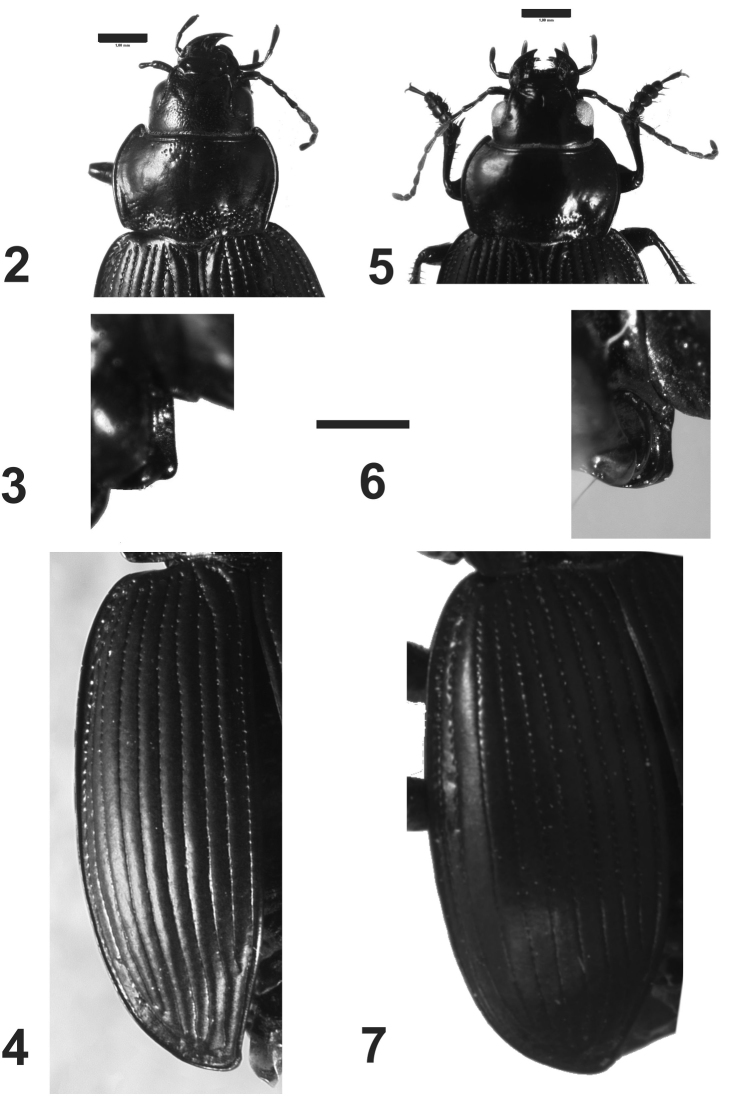
*Rhytidognathus ovalis*: **2** Head and pronotum, dorsal view (Scale = 1 mm) **3** Lateral view of prosternal apophysis (Scale = 1 mm) **4** Dorsal view of elytra **5** Head and pronotum, dorsal view (Scale = 1 mm) **6** Lateral view of prosternal apophysis (Scale = 1 mm) **7** Dorsal view of elytra.

**Figures 8–9.**
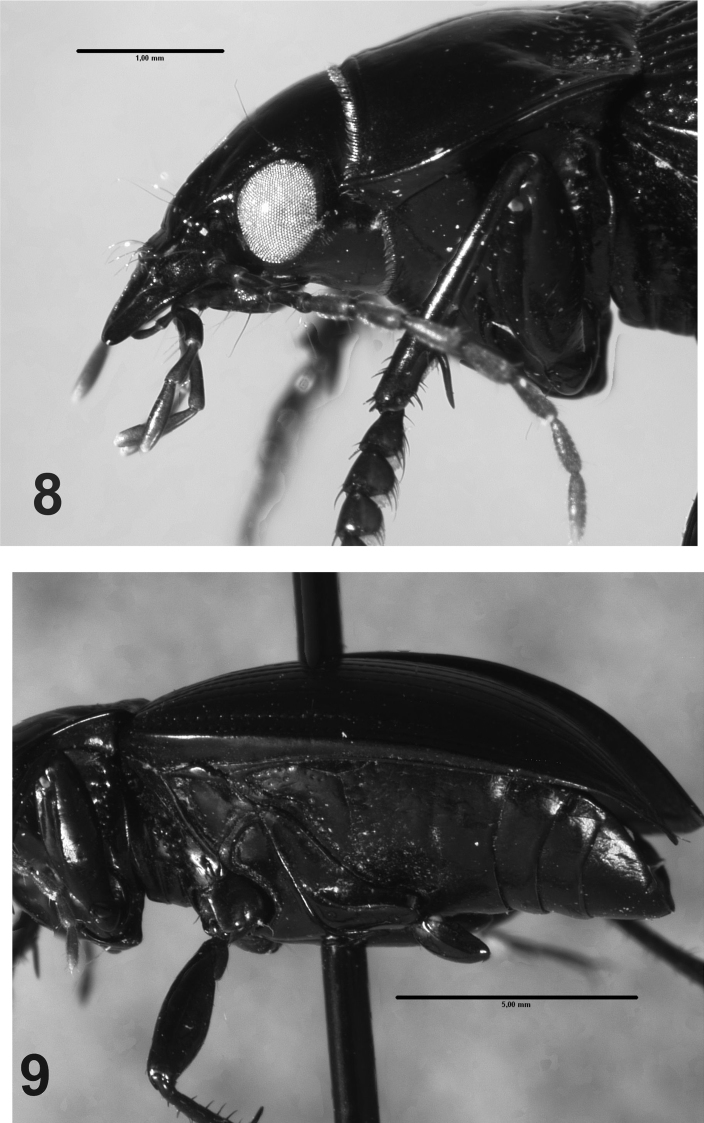
*Rhytidognathus platensis*: **8** Lateral view of head showing the eyes (Scale = 1 mm) **9** Lateral view of meso-metathorax and abdomen (Scale = 5 mm).

*Head*.Labrum short, transverse, bilobate at anterior margin; clypeus with two subparallel lateral sulci slightly developed, projected at the base of the frons (Figs 2, 5); mentum and submentum not fused, mentum with four setae, two lateral to the tooth, and two at the base; mentum-tooth bifid; glossa with a central carina, with two apical setae; glossa with two setae, paraglossae rounded, not projected; galea biarticulate, distal article as long as anterior one; mandibles with several dorsal transverse sulci; last maxillary and labial palpomeres long and truncate at apex; antennomeres three times as long as wide; antennae long, reaching the base of the elytra (Fig. 5); antennomeres fusiform, pubescent from the fifth antennomere (Fig. 8).

*Prothorax*.Pronotum wide, wider than head, with anterior angles projected forward (Figs 2, 5); median line slightly delimited; base of pronotum with strong punctures (Figs 2, 5); pronotum without setae on lateral margin; lateral margin rounded, without sinuosity, base bisinuate; prosternal apophysis with a longitudinal sulcus at apex, and a small protuberance or carina; prosternal apophysis projected posteriorly, but short, not touching the mesosternum, border of apophysis straight (Fig. 3) or concave (Fig. 6).

*Pterothorax*:mesoepisternum with deep punctures (Fig. 9); metaepisternum with a row of punctures and two apical sulci (Fig. 9); elytra twice as wide as than pronotum, without shoulders (Figs 4, 7), with borders rounded, elytra increasing in width to the apex, the widest part on apical third (Fig. 1); elytral epipleura more than twice wider at base than at apex, decreasing in width from base to apex; scutellar stria complete; striae with punctures, deep on the basal third, shallower on the second third and on apical third imperceptible, striae well delimited and deep all along their length (Fig. 4); setae only on ninth interval, with six or seven setae. Apterous.

*Legs*. Protarsomeres 1-4 and mesotarsomeres 1-3 of male with adhesive setae, wider than in females. Protrochanters with one seta present. Protarsomeres 2 and 3 of male wider than long; metatarsomeres long.

*Abdominal sterna*.Sterna III-V constituting more than two thirds of the length of abdomen; sulcus of separation of sterna III-IV and IV-V not reaching the center; female sterna VIII without apical sulcus, with two apical setae. Sternite III and IV with deep basal punctures.

#### Comparative notes.

The genus *Rhytidognathus* shares with *Pseudomigadops* Jeannelthe characteristic of having the elytral striae punctured and differs from it by having the articles of maxillary and labial palpi elongated and thin, as well as by having the mandibles carined dorsally. This last character is exclusive to the genus within the tribe.

##### Key for differentiating the species of *Rhytidognathus*

**Table:** 

1	Elytra oval, completely black; labrum black; elytral striae deep, interstriae convex (Fig. 4); superior border of eyes straight; prosternum with a median apical prolongation that projects dorsally (Fig. 3)	*Rhytidognathus ovalis*
–	Elytra more rounded, with interstria 8 reddish; eytral striae marked but not deep, interstriae flat (Fig. 7); labrum with lateral borders yellowish; upper border of eye rounded (Fig. 8); prosternum with a slight swelling in the apical region (Fig. 6)	*Rhytidognathus platensis*

### Rhytidognathus ovalis

(Dejean, 1831)

http://species-id.net/wiki/Rhytidognathus_ovalis

Nebria ovalis Dejean, 1831: 581.
Rhytidognathus ovalis : Chaudoir 1861.

#### Material.

Male and female,Cerro Colorado Uruguay, Florida(MLP); male Banda Oriental (IADIZA).

#### Diagnosis. 

Head with deep punctures in front, as well as at the base and apex of pronotum; elytra black, concolor; labrum concolor; legs black or dark red, tarsi reddish; apex of median lobe rounded.

#### Description.

Body shape oval. Length: 12–13 mm; coloration: black; with antennae light colored, reddish, and legs testaceous or dark reddish. Elytra black, concolor.

*Head*.Head with deep punctures in front, eyes slightly protruding, sub-quadrangular. Maxillary palpi black or dark red.

*Prothorax*. Wider than long, maximum width at middle (Fig. 2); dorsal surface with deep punctures at base and apex (Fig. 2); lateral margins narrow, curved; central longitudinal sulcus slightly developed; posterior transverse foveae impressed, with deep punctures (Fig. 2); prosternum with punctures; prosternal apophysis prolonged into a carina, which extends straight toward the dorsal region (Fig. 3).

*Pterothorax*:Elytra. Humeral angles rounded (Fig. 4); striae well impressed, and deeply foveate on basal third (Fig. 4), being less marked toward the apex; six to seven setae only in the ninth interval.

**Figures 10–18.**
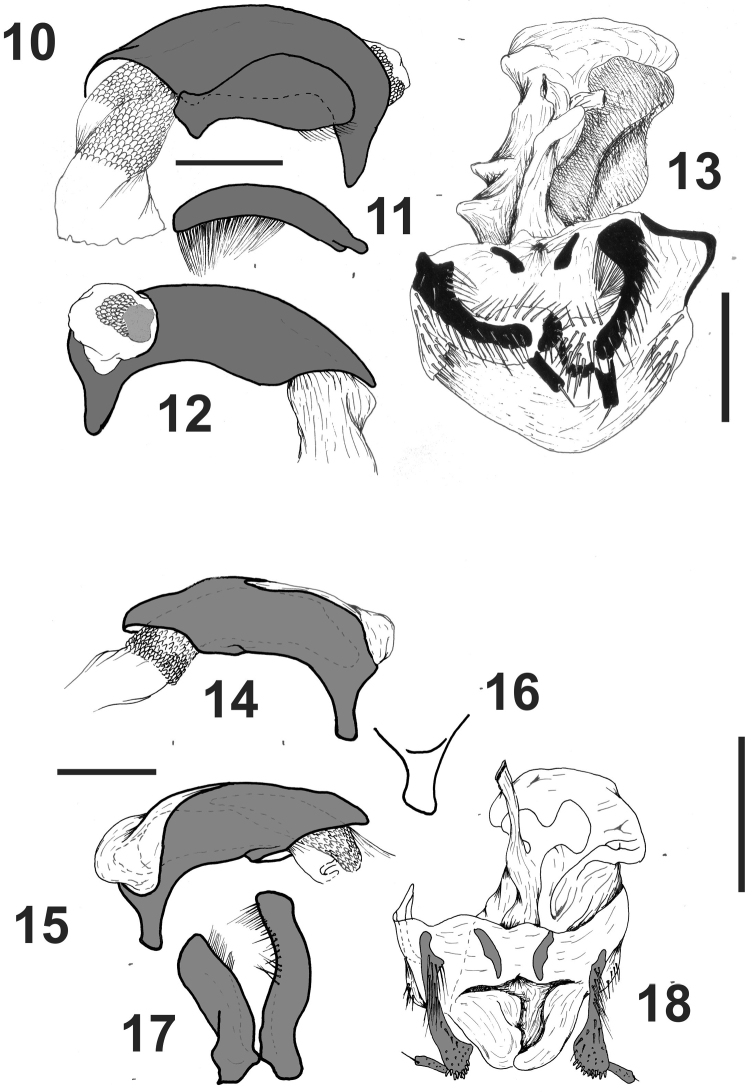
*Rhytidognathus ovalis*. **10** Median lobe and left paramere **11** Right paramere **12** Median lobe, right view **13** Female genital track, ventral view. *Rhytidognathus platensis*. **14** Median lobe, left view **15** Median lobe, right view **16** Apex of median lobe **17** parameres **18** female genital track, ventral view. Scale 1 mm.

*Male genitalia*(Figs 10–12). Median lobe wide, with apex rounded (Figs 10-12), apical orifice small, opening laterally to the right with a sclerified plate. Basal orifice wide, closed dorsally (Fig. 10), without basal keel. Left paramere wide with apex rounded (Fig. 11), with setae on apical third (Fig. 11). Right paramere straight and thin, the same width all along its length, with several setae from middle to apex (Fig. 11).

*Female genital track*(Fig. 13). With gonopod VIII small. Gonopod IX dimerous, the base with two sclerified plates, the apex small and without setae, with subapical setose organ (Fig. 13). Bursa copulatrix big, without accessory glands. Spermatheca on the base of oviduct, digitiform. Bursa copulatrix with a well developed sclerite.

*Intraspecific variation*. Jeannel (1938) found some intraspecific variation in the intensity of basal punctures of the pronotum and also in the coloration of the legs.

#### Distribution.

Uruguay: Montevideo: Montevideo (Chaudoir 1861). Florida: Cerro Colorado (MLP).

### Rhytidognathus platensis sp. n.

urn:lsid:zoobank.org:act:89A5BF3B-FB4B-4B75-95DA-D86FD0F667C8

http://species-id.net/wiki/Rhytidognathus_platensis

#### Type material.

Holotype: male, Argentina: Buenos Aires, Los Olmos (MLP); Paratypes, same date, one male two females (MELP, IADIZA); Entre Ríos(MACN), one female.

#### Diagnosis. 

Head with small punctures, on the borders; elytra black with interstria 8 reddish; labrum with the borders yellowish; interstriae flat; apex of median lobe sub-quadrangular.

#### Description.

Habitus as in Fig. 1. Length: 10.3 mm. Coloration: black; with antennae light colored, reddish, and legs testaceous, dark reddish. Labrum with borders yellowish; elytra black with interstria 8 reddish.

*Head*. Head with small punctures in front; eyes slightly protruding, rounded (Fig. 8). Maxillary palpi black or dark red.

*Prothorax*. Wider than long, maximum width at middle (Fig. 5); dorsal surface with punctures on the base (Figs 1, 5), apex with small or no punctures. Lateral margins narrow, curved; central longitudinal sulcus slightly developed; posterior transverse foveae slightly impressed. Posterior angles rounded. Prosternum without punctures or one or two on the apex. Prosternal projections not marginate, with a small apical tubercle, sinuate dorsally (Figs 6, 9).

*Metathorax*.Elytra with humeral angles rounded (Fig. 7); striae on basal third well impressed, and foveate, less impressed at apex. Ninth interval with six setae; elytral interval flat.

*Male genitalia*(Figs 14–17). Median lobe wide, with apex sub-quadrangular (Figs 14-16), apical orifice big, open dorsally and straight; basal orifice wide, closed dorsally (Fig. 14), without basal keel. Left paramere wide with apex rounded (Fig. 16), setae on apical third (Fig. 16). Right paramere thin, constricted in the middle, with setae from middle to apex (Fig. 16).

*Female genital track*(Fig. 18). With gonopod VIII small. Gonopod IX dimerous, the base with two sclerites, the apex small without setae, with apical setose organ (Fig. 18). Bursa copulatrix large, without accessory glands. Spermatheca on the base of oviduct, digitiform. Bursa copulatrix with a large sclerite.

#### Etymology. 

The name of the new species is related to the area where it was collected, La Plata district, near the La Plata river in Buenos Aires Province, Argentina.

#### Taxonomic considerations.

Tremoleras (1931) cited *Rhytidognathus ovalis* for Argentina. Tremoleras` specimen was held in his collection and now we can not find it. The description by Tremoleras (1931) does not allow a clear identification of this material. Roig-Juñent (2004) cited also *Rhytidognathus ovalis* for Entre Ríos province (Argentina), based on a female. In the presentcontribution, this female specimen is now considered as being *Rhythidognathus platensis*. Taking into account that *Rhythidognathus platensis* is distributed along the western shore of the La Plata river, we considered it more likely that Tremoleras` specimen belongs to the new species, *Rhythidognathus platensis*, and not to *Rhythidognathus ovalis*.

#### Distribution.

Argentina: *Buenos Aires*: San Isidro(Tremoleras 1931); Los Olmos (La Plata); *Entre Ríos*.

#### Habitat.

The new material was collected in the locality of Lisandro Olmos (La Plata, Buenos Aires) at “La Nueva Era” farm (35°01'18"S, 58°02'07"W) (Fig. 20), devoted to horticultural production under organic management (Fig. 21). The area has elevations of about 30 m, with soils derived from the Buenos Aires belt corresponding to grassland soils. It is surrounded by horticultural crops grown under cover and in the open, primarily tomato, pepper, leafy vegetables, celery, eggplant and small plots of corn, among others. Cut flower production in greenhouse conditions is also important in this area.

Samples were collected by pitfall traps set up in a 2000 m^2^-area cultivated with lettuce (*Lactuca sativa*), onion (*Allium cepa*), radish (*Raphanus sativus*), rocket (*Diplotaxis* sp.), cabbage (*Brassica oleracea)* and different types of weeds. This habitat has no native vegetation. Probably *Rhytidognathus platensis* inhabits the patches of semi-natural vegetation surrounding the crops. It has been proven that carabids move between cultivated and uncultivated patches (Marshall and Moonen 2002, Magura 2002).

On the shores of La Plata river in Buenos Aires province we found two natural habitats. One habitat is close to the river and includes: a) cliffs, with small forest of *Celtis tala* and other arboreal species, b) riparian shallows extending between the cliffs and the river and constituting a low plain that gets flooded, similar to the marshes of the Paraná river delta. The soil is clay and salty, and the vegetation is characterized by halophytic steppe with dominance of low grasses such as *Distichlis spicata*. The second habitat, the Pampean plain, lies above the cliffs. This lowland has a temperate climate, with an even year-round precipitation regime, soil type is loam, and the plants that dominate the landscape are herbs that compose the extensive Pampean grassland, a steppe. The typical original plant community comprises species of the genera *Stipa* and *Piptochaetium*. This landscape is accompanied on different sites by low shrubs of several species of *Bacharis*.

Predictive models of distribution show that the genus *Rhytidontahus* isrestricted to the coast and areas close to the La Plata river and the delta of the Paraná and Uruguay Rivers (Fig. 20), occupying shore habitats and the Pampean grassland near the shore. This Pampean plain has been strongly modified, allowing for great agricultural development with establishment of annual crops and pastures, leaving hardly any native vegetation in the region. The Pampean grassland and forest close to the La Plata river and to the high Paraná River differ in species and habitat conditions from the areas inhabited by nearly all sister groups of *Rhytidognathus*, the genera *Lissopterus* Waterhouse,* Migadopidius* Jeannel and *Pseudomigadops*. *Migadopidius* occupy temperate *Nothofagus* forests(Fig. 24, Table 1). *Lissopterus* and *Pseudomigadops* (Figs 22-23) occur in habitats closer to the shore, principally sub-Antarctic forest or moorlands (Figs 22–23, Table 1). The unique genus of the sister group inhabiting grassland is *Pseudomigadops*, in some part of Malvinas Islands. As we can see, *Pseudomigadops* inhabits coastal forest and grassland, like *Rhytidognathus*, but species composition in their habitats is far from being the same, as the former is of sub-Antarctic origin and the other of Neotropical origin (Morrone 2004). Climatic conditions are not the same either, and if we look at the variables that explain the predictive models of distribution of these four Migadopini genera, the most important variable is temperature (Table 1).

##### Biogeographic considerations

Because of its particular distribution pattern and its phylogenetic relationships with other tribes, the Migadopini have been used to explain some very different biogeographic views, such as an austral origin and separation by vicariance (Jeannel 1938, Brundin 1966) or a Holarctic origin, separate dispersal to the southern continents, extinction in tropical and subtropical regions (Darlington 1965). Beyond the different proposals regarding the origin of the tribe, everybody considers that its current restricted distribution is relictual (Jeannel 1938, Darlington 1965). Upon the advent of the theory of plates as applied to the continental drift, it was put forward that many groups with distribution patterns similar to those of migadopines be considered of austral origin, whose fragmentation led to their present distribution. By applying a Dispersal and Vicariance analysis, Roig-Juñent (2004) put both hypotheses to test and his conclusions concur with Jeannel’s saying that the tribe has had an origin in the southern hemisphere and that its current distribution across the southern continents has been due to vicariant events. Notwithstanding, the analysis yielded no support for the existence of three separate phyletic lines (monophyletic groups): Australian, New Zealander and American, as Jeannel proposed (1938). This shows that some clades would have originated before the fragmentation of some parts of Gondwana.

Regarding the present distribution of the Migadopini in South America, it is restricted to three disjunct areas. The first is in the Ecuadorian Andes, where the genus *Aquilex* occurs at about 4300 m elevation at Páramo (Moret 1989); the second is on the shores of the La Plata river where *Rhytidognathus* lives in Pampean grassland and riparian forest environments; and the third, which is the largest in surface area and coincides with the sub-Antarctic region in Chile and Argentina, includes all *Nothofagus* forests and sub-Antarctic regions up to Cape Horn. The latter is the area with highest number of Migadopini genera, and where most taxa show more phylogenetic affinity to other taxa from southern regions (New Zealand, Australia) than to those from the rest of the Neotropics. Although the present distribution of the Migadopini is largely restricted to the sub-Antarctic region in South America, it is likely that, at some point of the Cenozoic, the tribe may have had a broader distribution. The sub-Antarctic biota expanded to more northern areas and its later retraction left areas with relictual distributions. Such is the case of the Fray Jorge forests in Chile (30° 40´44” S, 71° 40´54” W) or the *Araucaria* forests in the south of Brazil and north of Argentina (26° 27¨S, 53° 37´W). This expansion might explain the presence of *Rhytidognathus* in the La Plata river because, being apterous and large-sized, this taxon has almost no capacity for dispersal. Moret (1989) considers the same situation for the genus *Aquilex*, which would have originated from its southern ancestors in the pulses of northward expansion of the sub-Antarctic biota during the Cenozoic.

Considering the particular distribution of *Rhytidognathus*, the biogeographic analysis carried out by Roig-Juñent (2004) shows that this genus would have been split by a vicariant event from its sister group (*Lissopterus* + *Pseudomigadops* + *Migadopidius*) which now inhabits the Magellanic region or the northern *Nothofagus* forests. Although the distance to the Magellanic region exceeds 3000 km and is 1000 kmto the *Nothofagus* forest region, the possibility of a vicariant event is feasible because, as mentioned for the austral region of South America, its cold austral biota experienced expansions during the Cenozoic whereby the genus came to occupy areas more northern than the current ones (Romero 1986, Barrera and Palazzesi 2007). So the separation of *Rhytidognathus* may have been caused either by vicariance or by isolation upon the southward retraction of the austral biota. Numerous are the relictual taxa than can be found in the Pampean region and south of Brazil, such is the case among carabids of the tribe Broscini.

In analyzing the environmental features of each genus, we find that there could also have been environmental features involved in the split. Figures 21–24 show the potential distribution range of *Rhytidognathus* and that of its sister genera. For these four genera, we find three clearly separate areas, one is austral sub-Antarctic, another one comprises the cold-temperate forests, and the third one encompasses the Pampean steppe and riparian forests along the La Plata river. The Pampean region is the exception with respect to the other habitats where migadopines occur in South America, and to the remaining circum-Antarctic regions, because most are from cold-temperate or cold environments, such as the species of *Loxomerus* Chaudoir (Johnson 2010). Although the Pampean grassland is a temperate area, it has warm summers and the vegetation is Neotropical in origin, not austral.

In other cases, it has been put forward that there often is niche conservation, commonly observed in species of the same genus whose potential distributions show areas occupied by other species of the genus rather than by them. However, we see that a shift has occurred among these four genera regarding the environment occupied by some of them. We propose that the environment occupied by the ancestor of *Rhytidognathus* and the sister group could have been cold-temperate coastal or riparian habitats, either forest or grassland (present in *Rhytidognathus* and *Pseudomigadops*). An arid barrier formed during the Cenozoic between the Pampean and sub-Antarctic regions (Barreda and Palazzesi 2007), isolating *Rhytidognathus*, and the current species of this genuswould have had to become adapted to this more temperate climate.

**Table 1.** Habitat characterization and the major variables explaining the predictive model of distribution obtained by Maxent.

	**Habitat**	**variables**
*Rhytidognathus*	Lowlands, 30-m altitude, in Pampean grasslands, and probably in riparian forests along the La Plata river and the Paraná river delta.	67.3%: Isothermality:17.0% :Precipitation Seasonality (Coefficient of Variation)10.1 Mean Temperature of Wettest Quarter
*Pseudomigadop*s	Lowlands, sea level to 10-meter altitude; in Malvinas grasslands (mainly of *Poa flabellata*) and Magellanic moorland (of *Empetrum rubrum*).In Navarino, southern Tierra del Fuego (near Beagle Channel), Isla de los Estados and Cape Horn *Nothofagus betuloides* forest on the coast and Magellanic moorland (*Empetrum rubrum*) (Niemela 1990)	46.9% Max Temperature of Warmest Month14.7 % Mean Temperature of Driest Quarter11.8 % Mean Annual Temperature
*Lissopterus*	Lowlands, sea level to 5-meter altitude; in Malvinas grasslands (mainly of *Poa flabellata*) and Magellanic moorland (of *Empetrum rubrum*).In Navarino, southern Tierra del Fuego (near Beagle Channel), Isla de los Estados and Cape Horn *Nothofagus betuloides* forest on the coast and Magellanic moorland (*Empetrum*) (Niemela 1990).Sub-Antarctic maritime areas including off-shore and more remote islands (Erwin 2011)	66.0% Max Temperature of Warmest Month11.9% Altitude6.1% Annual Temperature Range
*Migadopidius*	*Nothofagus* forest and *Araucaria* habitat; mixed forest (*Araucaria araucana*, *Nothofagus dombeyi*, *Nothofagus antarctica* and *Nothofagus pumilio*) (Dapoto et al.* 2005)*	63.0% Mean Temperature of Wettest Quarter29.0% Precipitation of Coldest Quarter

**Figures 19–20.**
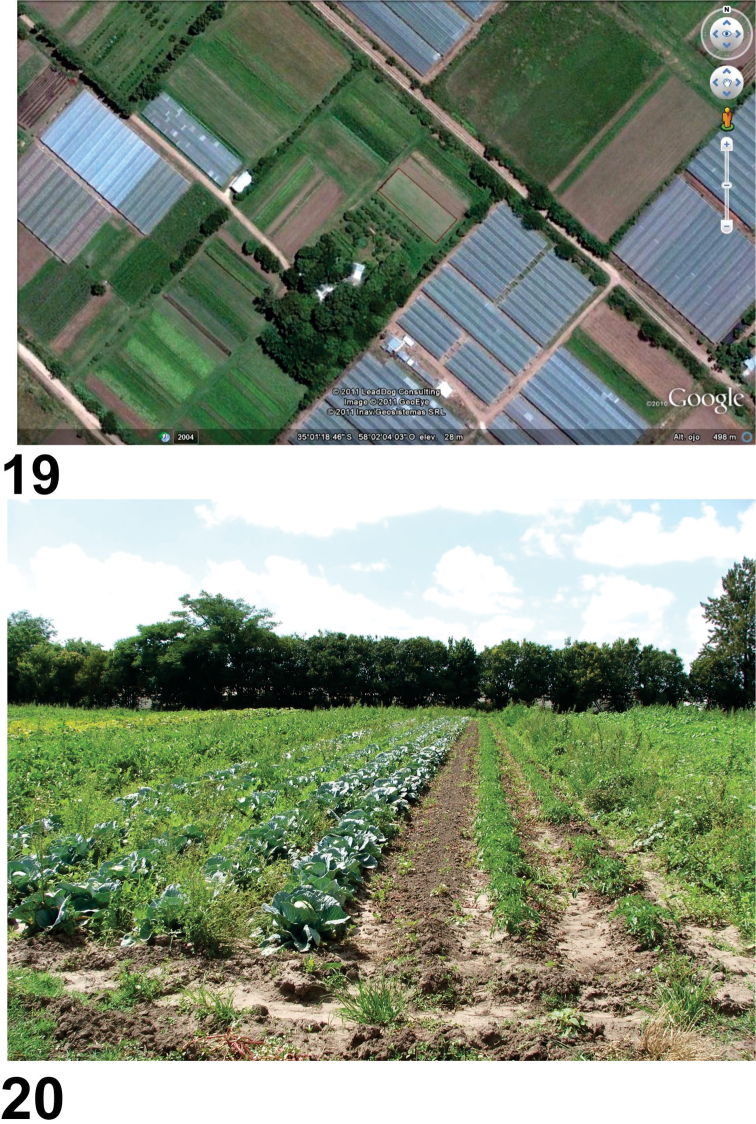
Habitat of *Rhytidognathus platenesis*. **19** Aerial view of the collecting area **20** Area where the study was developed, showing the crops.

**Figures 21–24.**
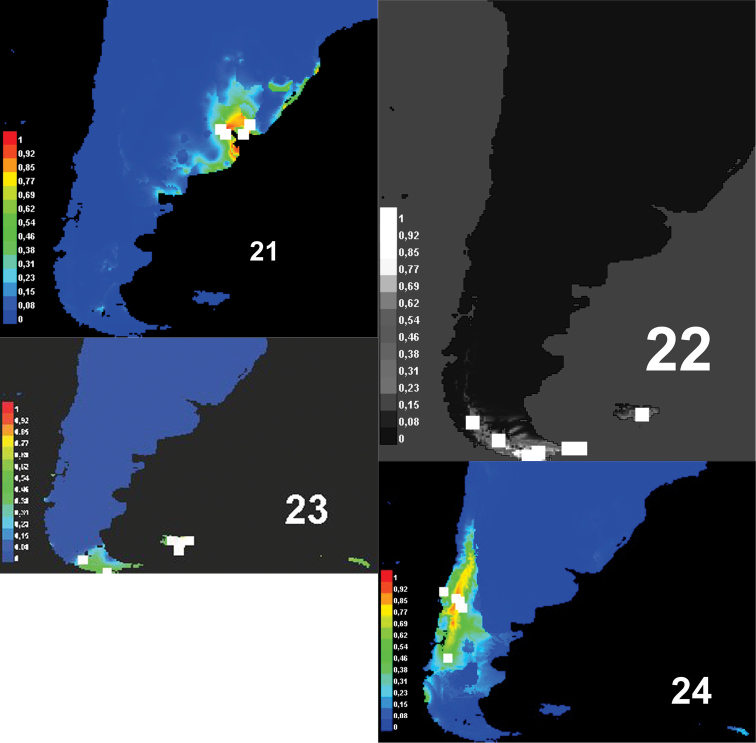
Potential distribution of: **21**, *Rhytidognathus* **22** *Pseudomigadops* **23** *Lissopterus* and **24** *Migadopidius*. Known localities are in white, probabilities of occurrence are indicated in different shades of grey.

## Supplementary Material

XML Treatment for Rhytidognathus

XML Treatment for Rhytidognathus ovalis

XML Treatment for Rhytidognathus platensis
